# WiFi Based Fingerprinting Positioning Based on Seq2seq Model

**DOI:** 10.3390/s20133767

**Published:** 2020-07-05

**Authors:** Haotai Sun, Xiaodong Zhu, Yuanning Liu, Wentao Liu

**Affiliations:** School of Computer Science and Technology, Jilin University, Changchun 130012, China; s88500860@163.com (H.S.); zhuxd@jlu.edu.cn (X.Z.); wentaolwork@gmail.com (W.L.)

**Keywords:** WiFi based positioning, seq2seq model, deep learning, trajectory

## Abstract

Indoor positioning technologies are of great use in GPS-denied areas. They can be partitioned into two types of systems—infrastructure-free based and infrastructure-dependent based. WiFi based indoor positioning system is somewhere between the infrastructure-free and infrastructure-dependent systems. The reason is that in WiFi based systems, Access Points (APs) as pre-installed infrastructures are necessary. However, the APs do not need to be specially installed, because WiFi APs are already widely deployed in many indoor areas, for example, offices, malls and airports. This feature makes WiFi based indoor positioning suitable for many practical applications. In this paper, a seq2seq model based, deep learning method is proposed for WiFi based fingerprinting. The model can learn from different length of training sequences, and thus can exploit the context information for positioning. The context information denotes the information contained in the sequence, which can help finding the correspondences between RSS fingerprints and the coordinate positions. A simple example piece of context information is human walking routine (such as no sharp turns). The proposed method shows an improvement with an open source dataset, when compared against deep learning based counterpart methods.

## 1. Introduction

Indoor positioning denotes the problem of positioning during Global Positioning System (GPS) outages. Although it has been extensively studied over the recent years, there is still a lack of a satisfactory overall solution. Nonetheless, many indoor positioning techniques are proposed for different applications. For example, in References [1,2], Inertial Measurement Units (IMUs) are adopted for collecting inertial data, which is processed for positioning derivation using the Pedestrian Dead Reckoning (PDR) algorithm [3] for emergency responses. As this type of methods does not need any infrastructures specially installed in the location areas, it is categorized as an infrastructure-free system. As opposed to infrastructure-free systems, there are infrastructure-dependent systems available, including the ultrasonic based Crickets system [4], the Radio Frequency Identification (RFID) systems [5], the Ultra Wide Band (UWB) based systems [6], and the infrared signal based Active Badge system [7].

WiFi based indoor positioning systems are somewhere between the infrastructure-free and infrastructure-dependent systems. The reason is that in WiFi based systems, Access Points (APs) as pre-installed infrastructures are necessary. However, the APs do not need to be installed specially for positioning purposes, because WiFi APs are already widely deployed in many indoor areas, for example, offices, malls and airports. As a matter of fact, WiFi based positioning is very suitable for many Location based Service (LBS) applications due to its low cost and enough accuracy [8]. WiFi based positioning rely on the Received Signal Strength (RSS), which can be represented as a vector of RSS values from nearby APs. Such vector is typically called a fingerprint, so that WiFi based positioning is typically referred to as WiFi fingerprinting. WiFi fingerprinting generally follows two procedures: the offline training process and the online positioning process. In the offline training process, the RSS fingerprints and their corresponding coordinate positions are collected to create the fingerprint map (FM). Then in the online positioning process, the recently collected fingerprints are matched against the RSS fingerprints in the FM. According to the matching results, the coordinate position is estimated. For WiFi based fingerprinting, further aiding input can be adopted for improving accuracy, for example, inertial readings [9], floor plan information [10], Bluetooth beacons [11].

However, in many cases, only WiFi information can be employed. Although sometimes inertial readings are available from many WiFi enabled devices such as smartphone, inertial readings are not perfect and can result in large errors. Therefore, there are a lot of methods for pure WiFi based positioning. The essence of WiFi based positioning is to learn a mapping relationship of the signal space and the coordinate space. Artificial Neural Networks (ANNs) are a good tool for such a task, because in theory, the network can approximate any function if no limit is put on the scale of the learning parameters. The offline training process for WiFi positioning can correspond to that of the training of the ANN, while the online positioning process can correspond to the forward recognition of the ANN. As a result, ANNs have been explored for WiFi based fingerprinting. In Reference [12], a Stacked Denoising Auto-encoder (SDA) is adopted for WiFi fingerprinting to cope with the fluctuating nature of the RSS measurements. In References [13,14], a Recurrent Neural Network (RNN) is adopted for the training model between the RSS fingerprints and the coordinate positions. These methods have the same overall structure but differs in detailed implementation. In Reference [15], a variational auto-encoder is adopted for estimating the positions and the fingerprinting map at the same time.

For methods which rely exclusively on RSS fingerprints, such as the aforementioned deep learning based methods, the context information is not efficiently utilized for positioning. The context information denotes the information contained in the sequence, which can help to find the correspondences between RSS fingerprints and the coordinate positions. The fundamentals of the Wi-Fi fingerprinting problem has interesting parallels with the problem of translation tasks in Natural Language Processing (NLP). The seq2seq model has been previously studied to solve NLP translation tasks, so it shows potential to tackle WiFi fingerprinting with the aid of context information as shown in the following examples. Example One: In WiFi based positioning, different coordinate positions can correspond to similar RSS fingerprints due to noises in certain cases. With preceding and behind RSS fingerprints, the correspondence to coordinate positions can be determined. This is similar to that in the task of translation, where a word may have multiple meanings until it is put in a sequence of words. Example Two: Some fierce change of adjacent RSS fingerprints (or special changes in RSS sequences) normally corresponds to certain coordinate positions. This can be considered as certain patterns hided in the context information, which can be learned from the sequences. However, these patterns can be more implicit than that of word sequences (phrases or sentences in the task of translation). In sentences, patterns including nouns normally followed by adjectives, consistent plural terms for nouns and verbs, and so on are somewhat more explicit.

As deep learning based WiFi positioning methods are not designed to use and learn sequence patterns, in this paper we propose a seq2seq based learning method for WiFi based fingerprinting. The foundation of the seq2seq model is also a RNN but has added more techniques to ensure sequence processing [16,17]. It is adopted here where a sequence of RSS measurements is the input and the sequence of the coordinate positions as the output. The seq2seq model is adopted here for the following reasons—(1) it is similar for the task of translation and thus convenient for adoption in another application; (2) it can adopt the information from the context, such as the mentioned sequence patterns; (3) the model can cope with the situation where sequences of different lengths are adopted as the input. The greatest advantage of the proposed method is that it is able to learn from the training sequences, which in general cases, are related to the context information. Therefore, the proposed seq2seq based method can enhance the positioning accuracy without any additional information (especially an indoor map). In the process of applying the seq2seq model, we have also proposed a dataset enhancement strategy to enable better training and have applied the attention mechanism in the model. The proposed method is tested adopting an open sourced dataset, the results show that the proposed method can actually improve the positioning accuracy compared with its deep learning based counterpart methods.

The remaining of the paper is organized as follows—Section 2 reviews related works, Section 3 describes the proposed method, Section 4 shows the experimentation and Section 5 presents the conclusion and future work.

## 2. Related Work

In this part, we review related works for WiFi based fingerprinting including the traditional methods and the recently proposed deep learning based methods. In the last part of this section, we also give a simplified introduction of the seq2seq model adopted in the proposed method.

### 2.1. Traditional WiFi Fingerprinting Methods

**Figure 1:**
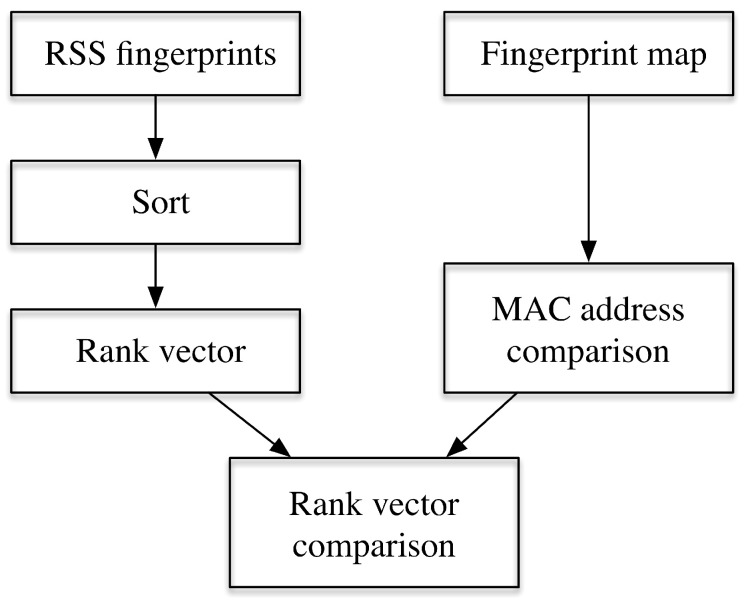
An implementation for calculating the rank based metric.

In traditional methods, it is assumed that the RSS vicinity [18] is kept in the coordinate space. Therefore the matching process is roughly based on the k-Nearest Neighbour (kNN) methods [19], where the estimated position is the weighted mean of the positions with k nearest neighbours in the RSS space. Traditional methods differ in terms of how they define the metric in the RSS space. Commonly seen metrics include Euclidean distance, Hamming distance, Lorentzian distance and Canberra distance. Here we show two classical distances (Equation (1) for the Lorentzian distance and Equation (2) for the Hamming distance):

(1) $$d=\sum_{i=1}^{n}\ln(1+|P_{i}-Q_{i}\left|\right)$$

(2) $$d=\sum_{i=1}^{n}\frac{|P_{i}-Q_{i}|}{P_{i}+Q_{i}},$$

where $P_{i}$ and $Q_{i}$ denote the two RSS fingerprints in matching. In Reference [20], the authors provide a comprehensive review of 49 different metrics adopted in WiFi based fingerprinting. Different metrics may have different features as shown in Reference [21], for example, the Euclidean distance has the best performance if two measurement points are close to each other; the Jaccard distance can achieve coarse estimation; however, it is sensitive to points far away from each other; the Spearman’s footrule performs especially well in the middle range. These metrics can be roughly categorized into—RSS value-based and rank-based. In the RSS value-based metrics, the RSS values are adopted while in rank-based metrics, only the relative magnitude information is adopted with information loss, which results in a faster calculation. Figure 1 gives the process for calculating rank based distance, where rank vector is generated according to the sorting values of the RSS values (from strongest to weakest). Then the ranks are assigned to the corresponding APs, whose identity is represented as their Media Access Control (MAC) address. Then in the online positioning process, the sorted rank vector can be compared with the vectors sorted in the fingerprint map.

### 2.2. Deep Learning Based WiFi Fingerprinting Methods

Deep learning is very suitable for WiFi based fingerprinting as the whole working procedure can cope with the actual WiFi fingerprinting procedures—the offline training and the online positioning. As more mature usages of deep learning techniques are available in the image processing field, it is also implemented in WiFi fingerprinting [21,22]. Here we list several methods for WiFi fingerprinting based on deep learning as follows.

**Figure 2:**
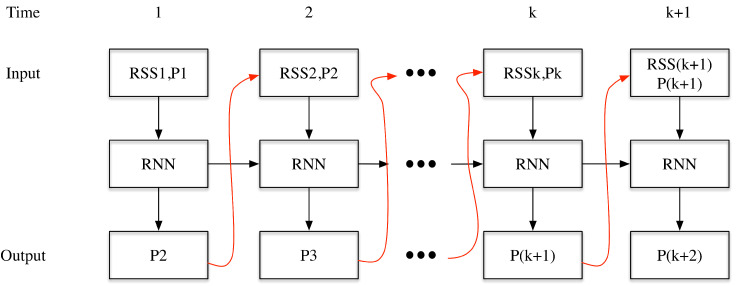
The Recurrent Neural Network (RNN) implementation for WiFi based fingerprinting in Reference [24], where the sequences of the fingerprints along with the positions are the input, and the sequences of the positions are the output.

- The authors of Reference [12] adopted a Stacked Denoising Auto-encoder (SDA) to model the relationship between the RSS fingerprints and the coordinates. The SDA can be adopted to pre-train the network without any need for labels, that is, the coordinate positions. The pre-training enables the network to be robust to the fluctuating noises in the RSS fingerprints. After pre-training, a few fingerprints with corresponding coordinate positions can be adopted to fine-tune the parameters in the network. This implementation enables semi-supervised training, where herein a lot of fingerprints are available while only a few has labels (coordinate positions).
- The authors in Reference [15] proposed adopting a variational auto-encoder to estimate the positions and the fingerprinting map at the same time. The advantage for using a Variational Auto-Encoder (VAE) in fact it contains a generation model, which can be adopted for generation of the RSS fingerprints after training. The results show that the usage of the VAE can improve the positioning accuracy compared with that of the method in Reference [23].
- Different from using a VAE and a SDA, the method in Reference [21] proposed using deep learning to learn the best metrics for WiFi fingerprinting. This method is also the framework of the traditional methods using kNN for positioning. However, through deep learning, some features between two RSS fingerprints are learned to be extracted, which is efficient for describing the vicinity in the coordinate space.
- Another type of methods relies on the RNN structure to model the positioning process. RNN is designed to enable finding data patterns in the time series. There are different RNN structures regarding to different input-output situations for WiFi fingerprinting. In Reference [24], as shown in Figure 2, a fixed length of positions and RSS sequences (f-RNN) are adopted as input, the sequences of positions are the output. The previous position estimation in time is adopted as the input to the next input. This network structure can enable the training process, however, for the positioning process, the initial position needs to be known prior to positioning. This renders the method not suitable in many cases.

The SDA method, VAE method and the f-RNN method are all aimed at learning the correspondences between the RSS fingerprints and the coordinate positions. They differ in the adopted deep learning models. These models endowed the methods with different learning features. For example, the SDA method is robust to noises, the VAE method can generate new RSS fingerprint map and the f-RNN are time series based. Instead of trying to find correspondence in the RSS space and the position space, the metric learning method aim to find the best metric in RSS space, which still rely on the traditional kNN model to achieve position estimation.

### 2.3. Basics of the seq2seq Models

**Figure 3:**
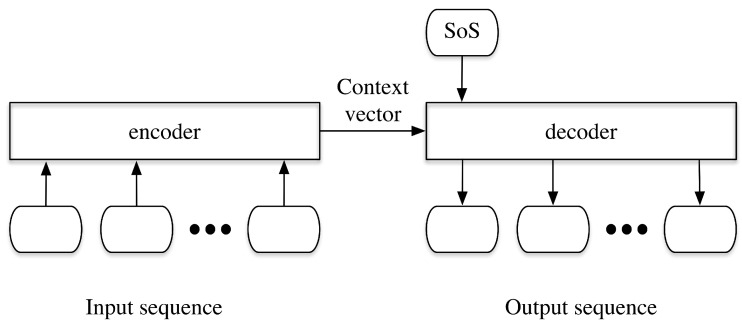
The encoder-decoder structure of the seq2seq model.

Seq2seq model is normally implemented in the NLP tasks, especially in translation. It is based on the encoder-decoder structure with sequences as the input and the output. The foundation of the model is the RNN network, especially the Long Short Term Memory (LSTM) cells. Information can pass through the cells. Therefore, LSTM cells can learn the patterns in time sequences. Here we give a simple introduction of how the seq2seq model is implemented in a normal translation task. The overall structure of the seq2seq model is shown in Figure 3. It is composed of an encoder and a decoder, both of which are with the RNN structure. The encoder uses a sequence as the input, and the hidden state of the last LSTM cell outputs a context vector, which can be considered as encoded information of the previous input sequences. Then the context vector along with the Starting of Sentence token (SoS) is adopted as the input to the decoder, which can produce the translated contents.

**Figure 4:**
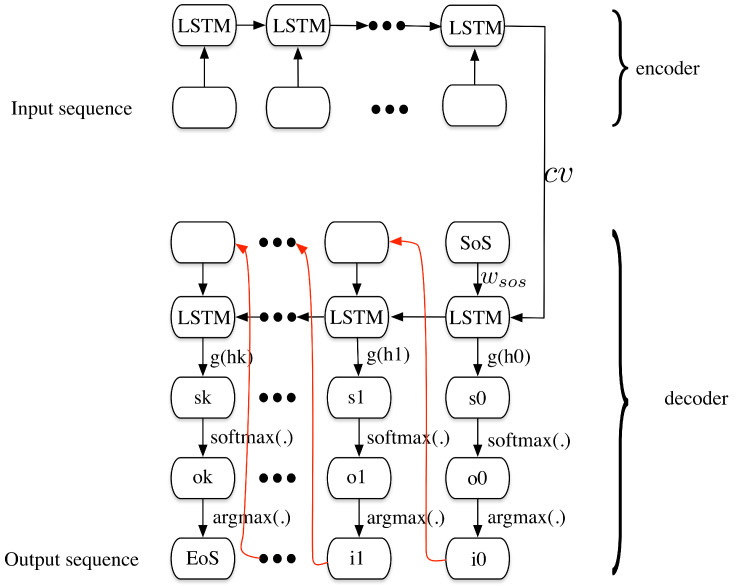
The detailed encoder-decoder structure of the seq2seq model.

Figure 4 shows the detailed information of the encoder and the decoder. In the encoder, the words represented by vectors (see word embedding in Reference [25]) are input to the LSTM cells. The stacked cell can output a context vector, which is the hidden state of the last cell. Herein it is represented as cv. Then in the decoder, cv along with the SoS token $w_{sos}$ is the initial input to the first LSTM cell. The output of the cells can be applied to some transformations, including a function mapping g(.), a softmax function softmax(.) to generate the word embedding probabilities $o_{0}$ in another language. Then the output of the previous cell goes through the function argmax(.) to get the most probable word embedding and is adopted as the input of the next one $i_{0}$. The decoder stops when the output of the last cell is the End of Sentence (EoS) token. The decoder process can be written as the following equations.

(3) $$\begin{array}{c} h_{0}=\mathrm{LSTM}\left(cv,w_{sos}\right)\\ s_{0}=g\left(h_{0}\right)\\ o_{0}=\mathrm{softmax}\left(s_{0}\right)\\ i_{0}=\mathrm{argmax}\left(o_{0}\right) \end{array}.$$

**Figure 5:**
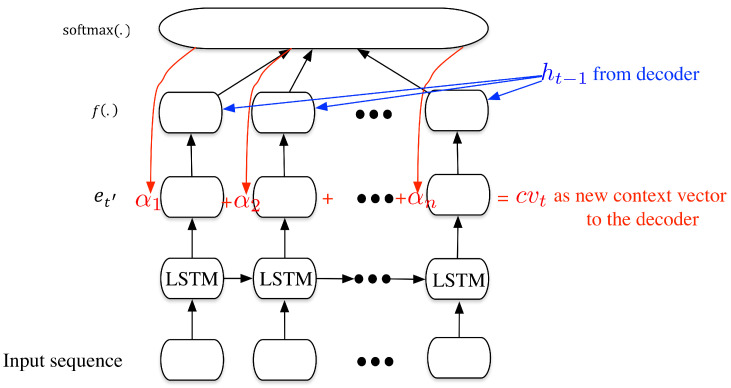
An illustration for the attention mechanism of the seq2seq model.

Sometimes if the input sequence is too long, the context vector may not be efficiently composed to encode the complete input sequence. One solution to that is to adding the attention mechanism to enhance translation performance, so that let the decoder informed with which word the translation corresponds to. The overall seq2seq model with attention mechanism is shown in Figure 5. In this model, the context vector is no longer the hidden state of the last cell in the encoder, it is a weighted sum of all the hidden state from previous states. The weights are jointly determined by the hidden states in the encoder and the previous hidden state in the decoder. The process of the seq2seq based processing with attention mechanism can be shown as following:

(4) $$\begin{array}{c} h_{t}=\mathrm{LSTM}\left(h_{t-1},\left[w_{i_{t-1}},cv_{t}\right]\right)\\ s_{t}=g\left(h_{t}\right)\\ o_{t}=\mathrm{softmax}\left(s_{t}\right)\\ i_{t}=\mathrm{argmax}\left(o_{t}\right), \end{array}$$

where $cv_{t}$ is the new context vector added to the input of the LSTM cells. The new attention vector is calculated as follows:

(5) $$\begin{array}{c} \alpha_{t^{\prime }}=f\left(h_{t-1},e_{t^{\prime }}\right)\in R\;\;\mathrm{for}\;\mathrm{all}\;t^{\prime }\\ \bar{\alpha }=\mathrm{softmax}\left(\alpha \right)\\ cv_{t}=\sum_{t^{\prime }=0}^{n}{\bar{\alpha }}_{t^{\prime }}e_{t^{\prime }}, \end{array}$$

where f(.) is a scoring function (possibly has different terms from Equation (4)), $e_{t^{\prime }}$ denotes the outputs of the cells in the encoder, $\bar{\alpha }$ is the weights determined by the hidden state $h_{t-1},$ from the previous LSTM cell in the decoder and the outputs from the encoder $e_{t^{\prime }}$. The new context vector $cv_{t}$ is the weighted sum of the outputs of the encoders.

## 3. The Proposed Method

The seq2seq model has features like sequence based processing, context learning and attention based processing. These features make it very suitable for WiFi based fingerprinting. The reasons are three folds.

- WiFi based fingerprinting is also a sequence based problem with the sequence of the RSS fingerprints as the input and the sequence of the coordinate positions as the output.
- In WiFi fingerprinting, the context information from the sequence can improve the positioning accuracy. Information like human walking patterns and floor plan constraint can be extracted from the sequences (though in the form of black boxes), which are helpful for enhancing positioning performance.
- The attention mechanism naturally suits WiFi based fingerprinting because each measured RSS fingerprinting corresponds to a certain coordinate position.

In both translation tasks and WiFi based positioning tasks, the input and the output are sequences. This inspires us to adopt the seq2seq model in WiFi based positioning with the input sequence as the RSS fingerprints and the output as the coordinate positions in the first place. Despite their similarities, NLP translation and WiFi based positioning have differences that must be addressed, including:

- The sequence space is discrete for translation while continuous for WiFi based positioning. In translation, a sequence of words denotes a discrete path. However, in WiFi based positioning, the RSS fingerprints (input sequence) and the coordinate positions (output sequence) are continuous in signal space and position space respectively.
- The context information (contained in sequences) matters more in translation than in WiFi based positioning. In translation, a word may have multiple meanings until it is put in a sequence of words. However, for WiFi based positioning, a RSS fingerprint normally only corresponds to a single coordinate position. This doesn’t mean that context information in RSS fingerprint sequence is unimportant. In some cases, different coordinate positions can correspond to similar RSS fingerprints due to noises. With context information, single correspondences can be identified.
- The patterns of sequence in translation are more explicit than that of WiFi based positioning. In translation, word sequences have many explicit patterns, for example, nouns are normally followed by adjectives. Normally inserting or deleting words randomly in a sentence can violate the patterns in the sentences. However, a RSS fingerprint sequence can still be meaningful if collecting more fingerprints in between. For RSS fingerprint sequence, the patterns are more implicit but still follow some patterns. For example, the fierce change of adjacent RSS fingerprints normally corresponds to certain coordinate positions.

To summarize, for WiFi based positioning task, the sequence space is continuous, with less explicit context information. As a neural network based learning model, the seq2seq model is well suited for WiFi based positioning, due to its ability to capture the context information and implicit patterns.

During the implementation of the seq2seq model to the problem of WiFi fingerprinting, some issues need to be solved.

- The RSS measurements are highly noisy and the length of the vector is not unified. Some pre-processing for the RSS measurements are necessary.
- The seq2seq model normally needs a lot of sequence samples for training. Therefore, in our implementation, some techniques are adopted for increasing training samples, that is, creating more sequences.
- Some modifications are added to the original seq2seq model, mainly including the issues about transforming the network from classification network to regression network, the EoS token and so on.

These three issues on the implementation of the seq2seq model are introduced in detail in the next three subsections.

### 3.1. Pre-Processing for the RSS Fingerprints

**Figure 6:**
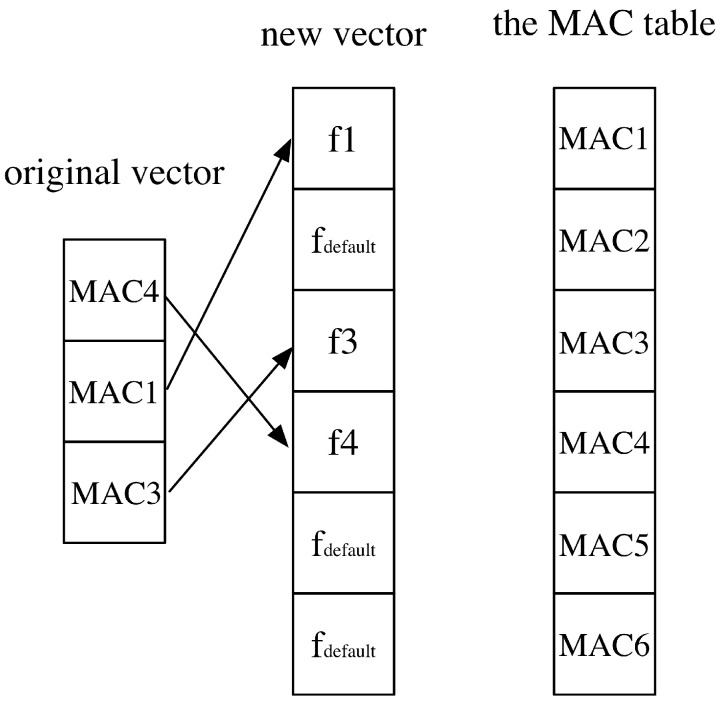
A simple example showing the aligning process of the RSS fingerprint. Here we assume that there are only 6 Access Points (APs). The original vector only has RSS values from MAC4, MAC1 and MAC3. The aligned new vector then has a vector length of 6 with the missing values as $f_{default}$.

The RSS measurements are severely affected by noise of different sources. The noise mainly comes from three sources: (1) fluctuations of the signal, sometimes a small distance change can bring a signification change in the RSS measurement. (2) noise due to the changing environment, for example, moving objects, different densities of people and so on. (3) measurement noise like thermal noise. Normally, devices like smartphones feature different measurements. As a result, the original RSS measurements can be very noisy, so that some smoothing process are needed to lower the effects of the noise. Besides the noise problem, other issues exist in the RSS measurements. For instance, the input to a neural network needs to have a constant dimension. However, the original RSS measurements have different dimensions because they may receive signals from different numbers of APs. We tackle the pre-processing issues as follows:

- The first step is to align RSS fingerprint vectors to the same length. Assuming that there are N collected fingerprints, and they can be represented from $F_{1}$ to $F_{N}$ respectively. In the collected fingerprints, $F_{1}$ to $F_{N}$ may have different length and each of the vector element may represent RSS values from different APs. From Equation (6), the union set of all RSS fingerprint is acquired. The function Set(.) returns the set of MAC of the fingerprints. Then according to the set $S_{AP}$, a table is established, where each index in the table corresponds to a specific MAC from an AP. Then according to the table, we align all the fingerprints shown in Figure 6. If a specific MAC in the original RSS vector is missing, then we fill the corresponding index with the value $f_{default}$. This value is assumed to be the noise level denoting that the signal from the AP is buried in noise. Herein the default value in our implementation is −110 dBm, which is also adopted in Reference [9]. After the processing, all the fingerprints now have the same length and can be adopted as the input to the neural network model.
  (6) $$S_{AP}=\cup \left\{Set\left(F_{1}\right),Set\left(F_{2}\right),...,Set\left(F_{N}\right)\right\}.$$
- As mentioned, the original RSS values has large noise due to signal fluctuations, dynamic environments and so on. Therefore, a process for lowering the noise is needed. From the previous step, we know that the missing RSS values are set to $f_{default}$. From the study in Reference [9], we know that the missing values also may due to the effect of noise. In the data collecting process, we know that fingerprints collected in recent times are also within nearby locations, resulting in similar RSS measurements. Based on that, here an iterative recursive weighted average filter is adopted. The descriptions of how and why the filter is needed is presented in detail in Reference [24].
- After the previous steps, we still need to tackle the heterogeneous distributions of the fingerprints as they can come from different devices. The problem that fingerprints from different devices have different distributions is noted as the device heterogeneity problem [26]. To render the collected distributions to be nearly equally distributed, a normalization process is added. We assume that the fingerprints vector coming from the same device can be still represented as $F_{1}$ to $F_{N}$. Then the normalization process can be presented as:
  (7) $$f_{normalized}=\frac{f_{raw}-f_{min}}{f_{max}-f_{min}},$$
  where $f_{raw}$ is the elements in the fingerprints before normalization, $f_{normalized}$ is the normalized RSS value, $f_{max}$ and $f_{min}$ are the maximum and minimum RSS values from the fingerprints $F_{1}$ to $F_{N}$ respectively. After the normalization, all the RSS values in the fingerprints are distributed between 0 and 1 regardless of which device they are collected from, as required by neural network methods.

After the three mentioned processing steps, the fingerprints are ready to be used as input to the neural network.

### 3.2. Input Data Augumentation

**Figure 7:**
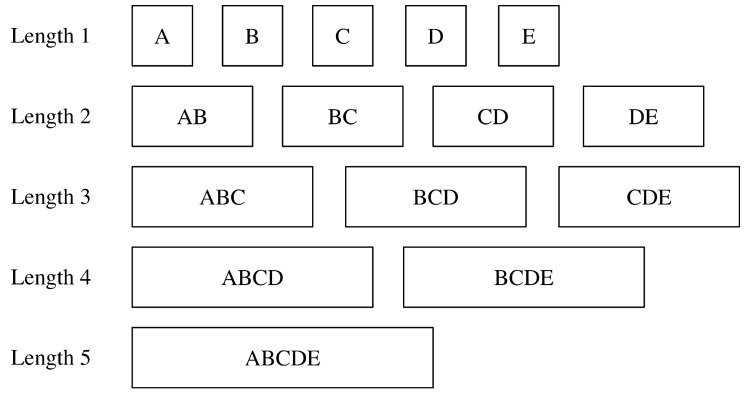
An example for increasing number of sequences through adding sliding window. In this case, the original trajectory has 5 fingerprints with ids of A, B, C, D and E respectively.

**Figure 8:**
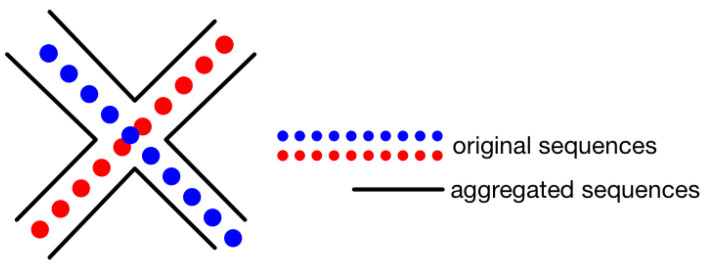
An illustration for sequence aggregating. After aggregating the original two sequences, 4 new sequences are generated.

The seq2seq model adopts a sequence as the input and normally needs a lot of training samples. In our implementation, it means we need to collect a large amount of trajectories with known positions and the corresponding RSS fingerprints in the online training phase. It needs a lot of effort for the data collecting prior to positioning, which can render the proposed method less applicable. To alleviate the shortage of the training samples, here we propose a strategy for increasing training samples, roughly comprised by the following tasks:

- We add sliding windows to the trajectories to provide training sequences with different lengths. This process is shown in Figure 7, where it is assumed that the initial trajectory has a length of 5. The ids of the fingerprints can be represented as A, B, C, D, E respectively. In this example, we can have 5 training sequences with length 1, 4 training sequences with length 2, 3 sequences with length 3, 2 sequences with length 4 and 1sequence with length 5. The total number of sequences for the single collected trajectory can reach up to 15. If the fingerprints in a single trajectory is *M*, then from adding sliding window, the number of sequences can be adopted is:
  (8) $$L_{slide}=\frac{(1+M)M}{2}.$$
- Another way to increase the number of training sequences is to inverse the original sequences. Also take the example from Figure 7, the inverted sequences are BA, CB, DC, ED, CBA, DCB, EDC, DCBA, EDCB and EDCBA. The number of sequences herein has increased by 10. In a more common situation with the number of fingerprints in a trajectory as M, the increased number of sequences due to inverting is:
  (9) $$L_{inverse}=\frac{(M-1)M}{2}.$$
- The number of sequences can be added through aggregating existing trajectories. This idea is inspired by the implementation in Reference [24], where single points are aggregated into sequences. Here we aggregate the sequences into new sequences. The idea is shown in Figure 8. We can see that in the figure, originally there are two trajectories with different colors of points showing the positions where the fingerprints are collected. In the circled area, some of the points from the trajectories are close. In this case, the two trajectories can be aggregated, producing another 4 new trajectories shown in black lines. Noting that the example presented in the figure is just an illustration. The aggregating process is presented in detail using the next 2 processes. Firstly, we calculate the distances between any two original trajectories. The distances denote any coordinate distance between one point in one trajectory and one point in the other trajectory. If a distance is under some distance threshold, then the two trajectories are referred to as potential trajectories to be aggregated. Then the points are referred to as critical points. We iterate this process to find out all the potential pairs of trajectories. Secondly, between any pair of potential trajectories, we take the critical points as shifting points from one trajectory to another and make new trajectories. If there are many critical points to shift, we shift the trajectories according to the following rule:
  (10) $$P\left(p_{i}\right)=\frac{1}{\sqrt{2\pi }\sigma^{2}}\exp\left(-\frac{d_{i}}{2\sigma^{2}}\right).$$
  Here, the $P(p_{i})$ denotes the probability of which point the trajectory is to shift, $d_{i}$ denotes the distances between the shifting points, $\sigma^{2}$ is the variance of the distribution. The rule implies that the nearer two points are, the more possible the trajectory should be shifted. According to the rule, a group of the critical points can add multiple new trajectories. In our implementation, we only take at most 4 new trajectories out of two original ones. In this way, new trajectories are aggregated from existing trajectories. As part of our contributions, we also propose to use the new trajectories for creating training sequences by using the sliding window and inverting processes.

### 3.3. Network Implementation

**Figure 9:**
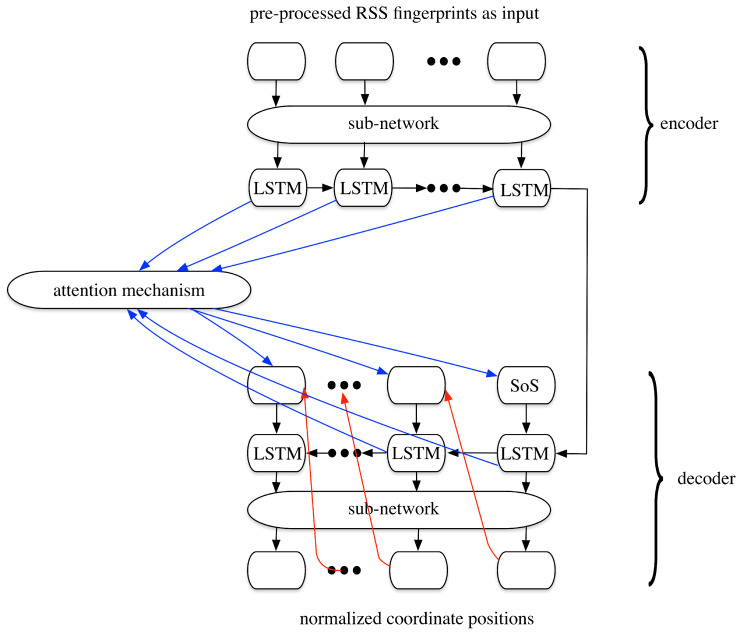
The network structure implemented in the proposed method.

The network implemented here is shown in Figure 9, which is similar to the seq2seq model. The differences are threefold.

- The output network for the prediction of positions is a regression structure other than the normal classification structure, thus no softmax function is applied to the output. From the network structure, we can see that there are no softmax functions in the decoder before the output. However, before the output, a function is needed to transform the output of the LSTM cells to a two-dimensional value representing the estimated coordinate positions. Noting that here, the coordinate positions should also be normalized as:
  (11) $$p_{normalized}=\frac{p_{raw}-p_{min}}{p_{max}-p_{min}},$$
  where $p_{normalized}$ can either be the x-axis or the y-axis.
- The length of the input sequences and the output sequences are the same in any situations, thus no EoS tokens are needed in our implementation. This makes the network easier to train than the normal seq2seq model.
- As both the input (pre-processed RSS fingerprints) and the output (the normalized coordinate positions) can present the corresponding metrics in their own forms, so no processes like word embedding are needed. However, in the encoder, before the normalized RSS fingerprint is input to the LSTM cells, a function or a small CNN network is added, which can downsize the dimension to input to the LSTM and makes our model easier to train. Noting that here the sub network shares the same weights for different RNN epochs in the encoder. This is the same for the sub network in the decoder.

Our network has adopted the Mean Square Error (MSE) during training shown as follows:

(12) $$\mathrm{loss}=\sum_{i=1}^{N}\sum_{j=1}^{O}\left[{\left(x_{i,j}^{pred}-x_{i,j}^{train}\right)}^{2}+{\left(y_{i,j}^{pred}-y_{i,j}^{train}\right)}^{2}\right],$$

where the number *N* denotes the number of training sequences, *O* denotes the number of positions in one training sequences, $x_{i,j}^{pred}$ along with $y_{i,j}^{pred}$ denote the predicted *x*, *y* axis positions by the network and $x_{i,j}^{train}$ along with $y_{i,j}^{train}$ denote the *x*, *y* axis positions in the training sequences.

Noting that in the proposed method, our contribution mainly lies in training data preparation (data enhancement) and network implementation. The data pre-processing, including pre-filtering of data and normalization are just typical and classical handle of data regardless of which method is adopted.

## 4. Experiments

### 4.1. Experimental Setup

**Figure 10:**
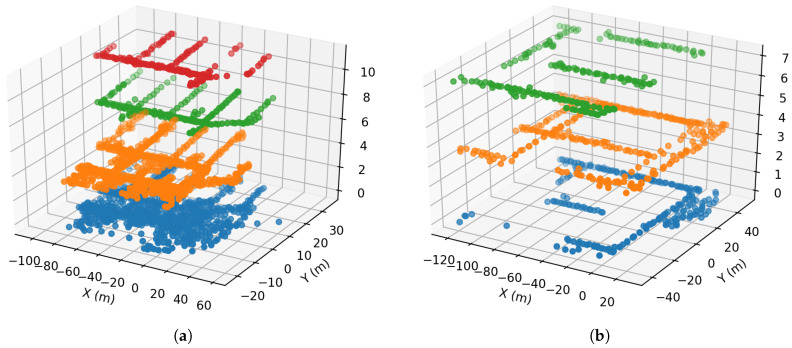
The geometric positions of fingerprints in (**a**) Building One and (**b**) Building Two. (The dataset is from Reference [27]).

To evaluate the accuracy of the proposed seq2seq based method, we perform the experiments adopting open-sourced WiFi positioning dataset [27]. This dataset is very suitable for testing the proposed method because the dataset is arranged in the form of trajectory sequences. In this form, the proposed method can effectively adopt the context information in the sequences to aid positioning. The dataset is collected in a campus containing two different buildings. The sampling frequency in the dataset is three fingerprints per second. The geometric positions showing where the RSS fingerprints are collected can be seen in Figure 10. We can see that the positions are 3-dimensional and the positions are distributed more densely in Building One than in Building Two. In this dataset, there are 354 different MAC addressed in Building One and the number for Building Two is 309. The dataset can be found in publication [27].

Note that here, the data in two buildings have different features, which can make the positioning accuracy in two buildings different. The positioning area in Building One is larger than that of Building Two, which means that Building One has a larger error bound. The samples in Building Two are in several batches, where the batches are far away from each other. This is similar to conduct positioning in many smaller areas, which reduces the positioning error.

In our implementation, we have adopted a 50% to 50% split for learning and testing using the enhanced dataset. The approach was implemented in Python using Tensorflow as the deep learning architecture. It is implemented in a x86 platform instead of actually running on mobile device. For the testing process (only forward estimation), the average running time for each positioning estimation is about 4.8 ms. We have repeated the experiments for 10 times. The showing results are the mean value of the ten times.

### 4.2. Accuracy of the Proposed Method

In the implementation of the proposed method, we have added some processes for enhancing the positioning accuracy. These processes includes:

- Adopting an iterative recursive weighted average filter to process the collected RSS to lower the noise, especially the fluctuations. This is a standard process for a wide range of WiFi positioning methods.
- Adding the attention mechanism to the model to help identify the correspondences between the current RSS fingerprint and the current geometric positions.
- Enhancing the dataset by cutting, inverting and aggregating the trajectories. This can render the neural network be trained with more sufficient data.

In this section, we perform experiments showing how these factors will affect the performance of the proposed method respectively. Here we consider the aspects of filtering RSS, adding mechanism and enhancing dataset as independent variables. In order to compare the effects of one variable, we make sure the other two variables are the same. For example, in the CDF curve comparisons of “adopting filtered RSS” and “adopted unfiltered RSS”, we mean that this CDF is acquired with attention mechanism and with dataset enhancement.

**Figure 11:**
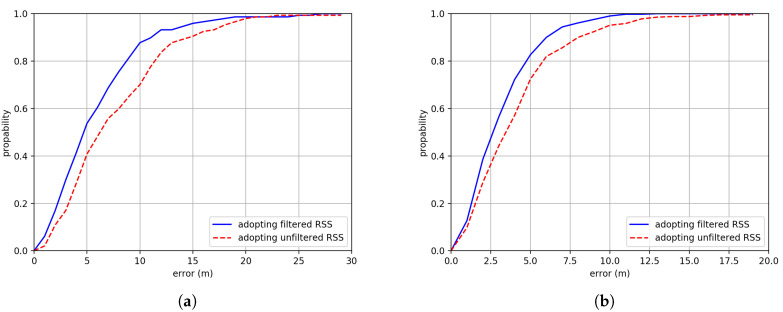
The Cumulative Density Function (CDF) comparison between adopting filtered RSS and unfiltered RSS in (**a**) Building One and (**b**) Building Two.

Figure 11 shows the positioning error of Cumulative Density Functions (CDFs) comparison between implementation adopting filtered RSS and unfiltered RSS. We can see that with filtered RSS, the positioning accuracy is better than that without filtering. The average accuracy has increased by about 1.75 m and 1.01 m adopting filtered RSS in Building One and Building Two respectively.

**Figure 12:**
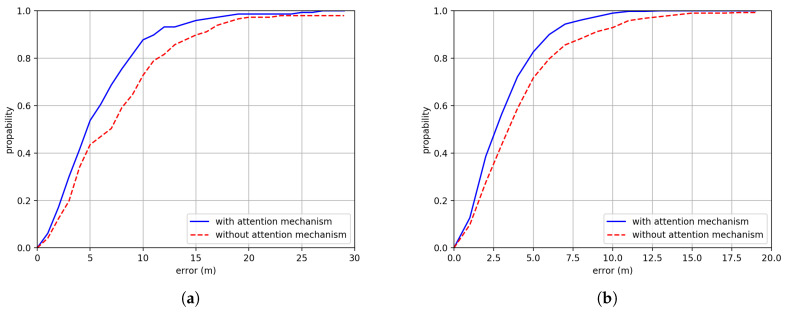
The CDF comparison between with attention mechanism and without attention mechanism in (**a**) Building One and (**b**) Building Two.

The effects of the attention mechanism is shown in Figure 12. We can see that adding attention mechanism can significantly increase the positioning performance in both Building One and Building Two. The positioning accuracy has increased by about 2.01 m and 1.13 m in Building One and Building Two.

**Figure 13:**
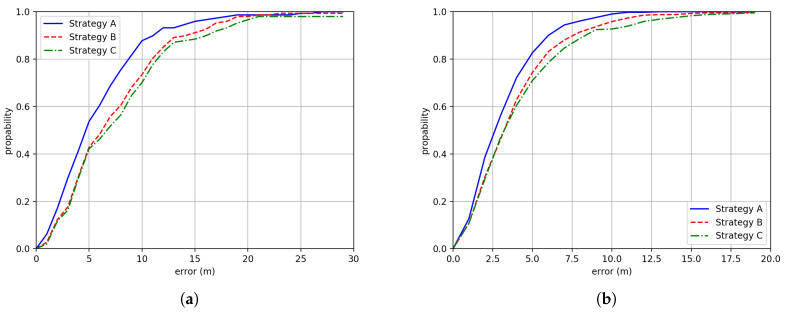
The CDF comparison between adopting different dataset enhancement strategies in (**a**) Building One and (**b**) Building Two.

Figure 13 gives the CDF comparisons between different dataset enhancement strategies. Three strategies are compared here:

- Strategy A: Adopting trajectory cutting, inverting and aggregating.
- Strategy B: Adopting only trajectory aggregating to generate new trajectories.
- Strategy C: Adopting only trajectory cutting (i.e., adopting a sliding window to vary the length of the trajectory described in the method section) and trajectory inverting.

Note that here, the results of no dataset enhancement are not shown. The reason is that we found the neural network cannot reach convergence without any form of dataset enhancement. From Figure 13, we can see that the strategy A has the best performance. Table 1 gives the summary of how the mentioned processes can enhance the performance of the proposed method. We can see that these processes are very effective in increasing the positioning accuracy, especially the proposed dataset enhancement strategy, which can make the neural network to converge in training. Noting that in the results of Figure 12 and Figure 13, we have adopted our proposed data enhancement Strategy A for the rest of the experiments, that is, including trajectory cutting, inverting and aggregating.

### 4.3. Accuracy Compared with Other Methods

We have compared the proposed method with the following methods:

- The classical PLGD method. This method is proposed in Reference [27], which adopts the structure of hierarchical positioning. The fingerprints are clustered as several sub-regions. In the positioning procedure, the potential regions are firstly narrowed to several sub-regions coarse positioning and then comes the fine positioning. This method have adopted several techniques such as PLGD metric, hierarchical positioning to increase accuracy, thus can be considered representative of the classical method without deep learning.
- The SDA method proposed in Reference [12]. The method adopts a SDA to cope with the fluctuating nature of the RSS fingerprints.
- The f-RNN based method proposed in Reference [24]. The method also relies on a RNN network, however the length of the RNN is fixed.

During the comparison of methods, we implemented the other methods and the shown results were produced by ourselves. For the method of PLGD, the codes were open sourced, we adopted their source code directly to reproduce their results. For the method of SDA and the method of f-RNN, we implemented them according to the descriptions in their respective publications. Also, during the implementation of other methods, pre-filtering was implemented. In fact, pre-filtering has now become a typical data preprocessing step independent of which method is adopted. However, for other strategies, such as adding the attention mechanism and adding dataset enhancement, they are considered part of our method and are thus not implemented in the comparison methods.

**Figure 14:**
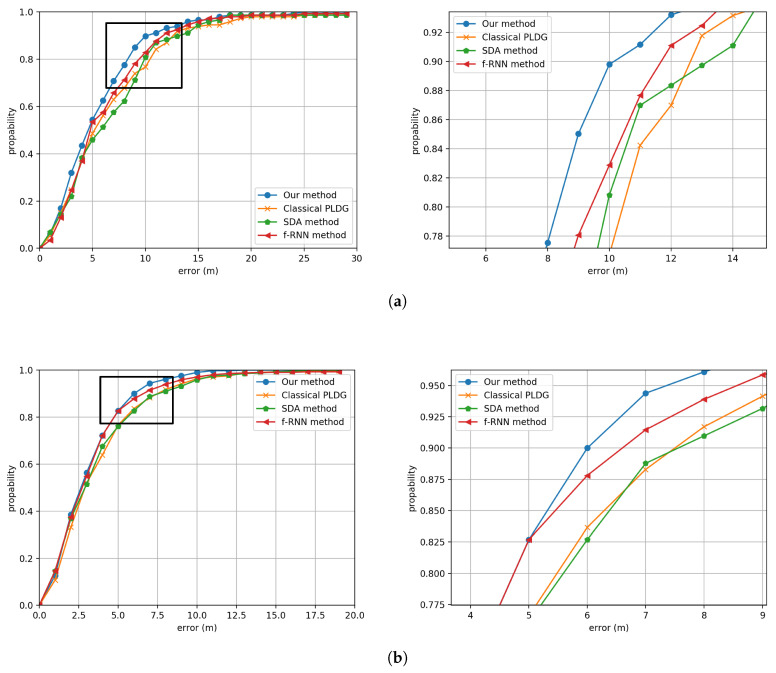
The CDF comparisons between the proposed method, the classical PLGD method, the SDA method and the f-RNN method in (**a**) Building One and (**b**) Building Two. Noting that here we enlarge the partial CDF for clarifying.

The CDFs of Building One and Building Two are shown in Figure 14. We can see that the proposed method has the best positioning accuracy among these methods. Table 2 gives a summary of the average positioning error for our method, the classical PLGD method, the SDA method and the f-RNN method. We can see that compared with PLGD method, SDA method and the f-RNN method, our method has increased the accuracy by 1.32 m, 1.27 m and 0.79 m in Building One, and 0.60 m, 0.51 m and 0.25 m in Building Two respectively.

## 5. Conclusions and Future Work

A seq2seq model based method is proposed in this paper for WiFi fingerprinting. In the proposed method, a sequence of RSS measurements is the input and the sequence of the coordinate positions is the output. After training, adopting the sequences, our method can learn the hidden information in the sequences, which can enhance positioning accuracy. In addition to the proposed method, we also proposed several processing techniques including the pre-filtering of RSS measurements, the attention mechanism and dataset enhancement (using trajectory cutting, inverting and aggregating). The proposed method is tested using open-sourced WiFi positioning dataset, the results show that:

- The proposed processes in our method, including pre-filtering of RSS measurements, attention mechanism and dataset enhancement can effectively increase the positioning accuracy. Particularly, the dataset enhancement strategy process, makes the network converge faster.
- Compared with other methods, including the classical PLGD method (no deep learning), SDA method and f-RNN methods (with deep learning), our method can sufficiently learn from the context information in the sequences and has an positioning accuracy increment of 1.32 m, 1.27 m and 0.79 m in Building One; 0.60 m, 0.51 m and 0.25 m in Building Two respectively.

Our future works are mainly in two folds.

- Studying the detailed effects of more hyperparameters. For example, the window length for positioning. We will be able to dig into this matter when we have collected new dataset where we can further change window length.
- Adjusting our proposed approach to be executed on an actual mobile device to test its performance, particularly the positioning latency.

## Figures and Tables

**Table 1:** Summary of the positioning accuracy enhancement adopting the filtered RSS, attention mechanism and dataset enhancement.

Accuracy Improvement	In Building One (m)	In Building Two (m)
adopting filtered RSS	1.75	1.01
adopting attention mechanism	2.01	1.13
dataset enhancement (Strategy A vs. B)	1.50	0.75
dataset enhancement (Strategy A vs. C)	2.36	1.06

**Table 2:** Average positioning error comparisons between the proposed method, the classical PLGD method, the SDA method and the f-RNN method.

Average Error	In Building One (m)	In Building Two (m)
Our method	5.50	3.08
Classical PLGD method	6.82 (1.32 higher)	3.68 (0.60 higher)
SDA method	6.77 (1.27 higher)	3.59 (0.51 higher)
f-RNN method	6.31 (0.79 higher)	3.33 (0.25 higher)
